# Supplementary material for: Electron Phase Detection in Single Molecules by Interferometry
Source: J Am Chem Soc. 2025 Jun 16;147(26):22572–9. doi: 10.1021/jacs.5c03056 (PMC12232316; doi:10.1021/jacs.5c03056)
Supplement: Supplementary file 1 [file ja5c03056_si_001.pdf]

Supporting Information:
Electron Phase Detection in Single Molecules by Interferometry

Zhixin Chen[1,*], Jie-Ren Deng[2], Mengyun Wang[1], Nikolaos Farmakidis[1], Jonathan Baugh[3], Harish Bhaskaran[1], Jan A. Mol[4], Harry L. Anderson[2], Lapo Bogani[1,5,*], and James O. Thomas[1,4,*]

[1]Department of Materials, University of Oxford, Parks Road, Oxford, OX1 3PH, UK

[2]Department of Chemistry, University of Oxford, Chemistry Research Laboratory, Oxford, OX1 3TA, UK

[3]Institute for Quantum Computing, University of Waterloo, Waterloo, ON N2L 3G1, Canada

[4]School of Physical and Chemical Sciences, Queen Mary University of London, London, E1 4NS, UK

[5]Departments of Chemistry and Physics, University of Florence, Sesto Fiorentino, 50019, Italy

Contents

Supplemental Section 1 Fabrication 2

Supplemental Section 2 Synthesis 4

Supplemental Section 3 Transport Measurement 5

Supplemental Section 4 Data Analysis 10

Supplemental Section 5 Simulation 12

Supplemental Section 6 Schematic Explanation 14

Supplemental Reference 15

Supplemental Section 1 Fabrication

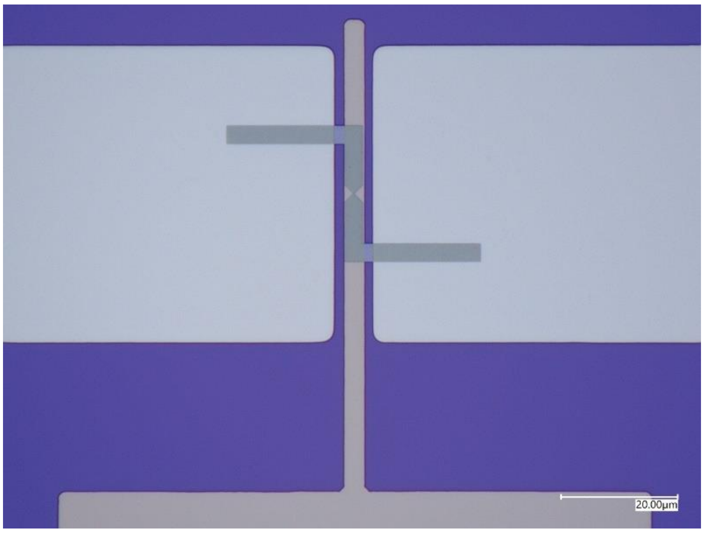

Fig. S1-1. Example optical image of the Al protected device; fabrication described in the Experimental section. The Z-shaped graphene tape with bow-tie centre was protected by a 50 nm thick layer of aluminium.

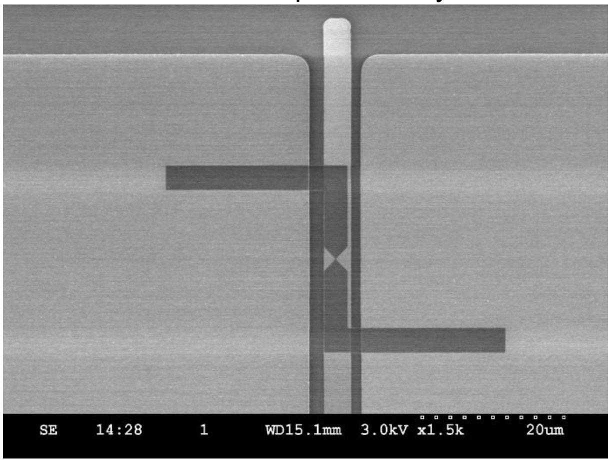

Fig. S1-2. Example SEM image of the aluminium-protected device.

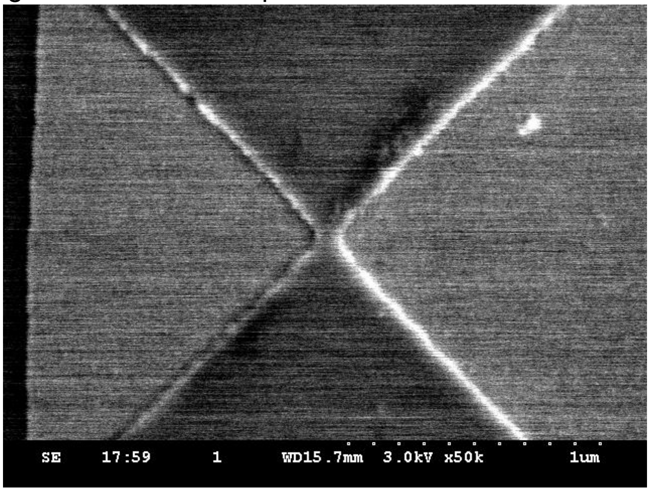

Fig. S1-3. Example SEM image of the aluminium-protected device (zoom-in view of the bow-tie shaped structure).

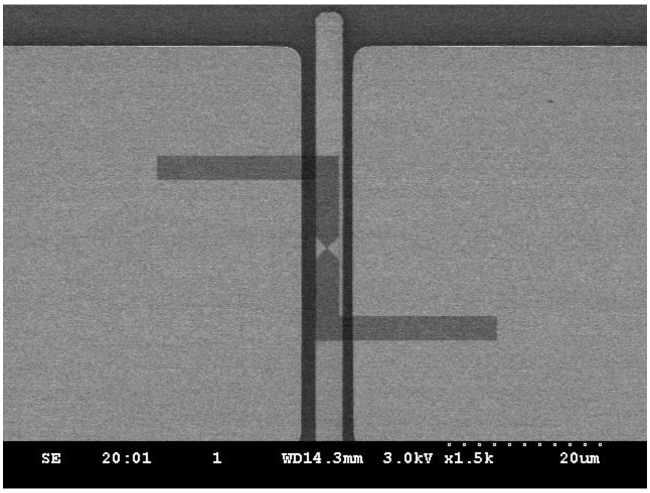

Fig. S1-4. Example SEM image of the device (the aluminium and residual PMMA have been removed).

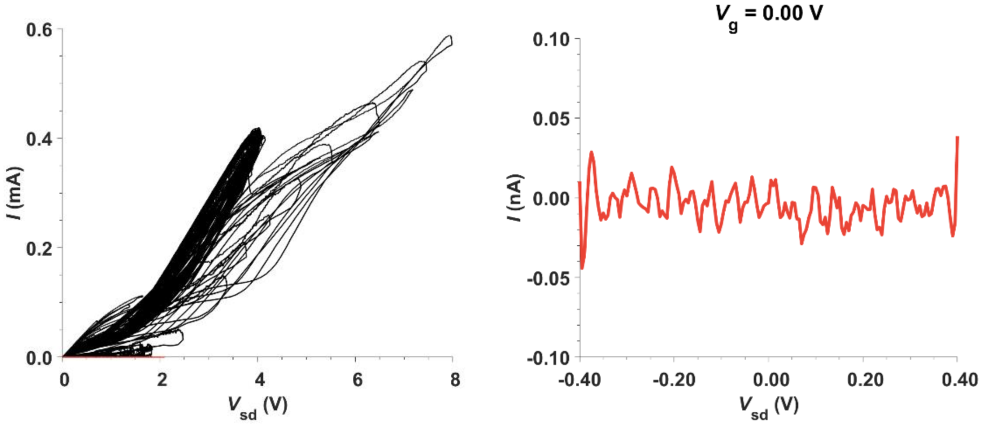

Fig. S1-5. Typical feedback-controlled electroburning traces to get a graphene nanogap (left) and I-V_{sd} trace after electroburning. Data is taken from device 1.

Supplemental Section 2 Synthesis

General Procedures

All reagents were purchased as reagent grade and used without further purification. Solvents for column chromatography were used in HPLC grade. Dry toluene and diisopropylamine (DIPA) were obtained from the solvent drying system MBraun MBSPS-5-BenchTop under nitrogen atmosphere (H_2O content < 20 ppm as determined by Karl-Fischer titration). Flash column chromatography was carried out using SiO_2 (60 Å, 230 – 400 mesh) under positive pressure. Analytical thin-layer chromatography was carried out on aluminium-backed silica gel 60 F254 plate. Evaporation in vacuum was performed at 25 – 80 ºC and 900 – 10 mbar. Size exclusion chromatography (SEC) was carried out using Bio-Rad Bio-Beads S-X1 (40 – 80 µm bead size). Semi-preparative gel permeation chromatography (GPC) was carried out on a Shimadzu recycling GPC system equipped with a LC-20 AD pump, SPD-20A UV detector and a set either of JAIGEL 3H (20 × 600 mm) and JAIGEL 4H (20 × 600 mm) columns using toluene/pyridine (99:1) as eluent at a flow rate of 3.5 mL/min. Reported yields refer to spectroscopically and chromatographically pure compounds that were dried under high vacuum (0.01 – 0.005 mbar) before analytical characterization.

NMR spectra were recorded at 298 K using a Bruker NEO 600 with a broadband helium cryoprobe, or a Bruker AVIII 700 with an inverse TCI $^{1}H/^{13}C/^{15}N$ cryoprobe. ^{1}H NMR spectrum is reported in ppm; coupling constants are given in Hertz, to the nearest 0.1 Hz. The solvent used was $CDCl_3$ which was calibrated to residual $CHCl_3$ at 7.26 ppm. Multiplicity (s = singlet, d = doublet, t = triplet, q = quartet, m = multiplet) and coupling constants were reported whenever possible. ^{1}H NMR signals were assigned based on comparison between compounds, chemical shifts, integrals and coupling constants.

MALDI-ToF spectra were measured using a Bruker MALDI Autoflex Speed spectrometer utilizing *trans*-2-[3-(4-*tert*-butylphenyl)-2-methyl-2-propenylidene]-malononitrile (DCTB) as matrix.

UV-vis-NIR absorbance measurement was recorded with a Perkin-Elmer Lambda 20 spectrophotometer or a Jasco V770 spectrophotometer using an Infrasil® Quartz 1 cm cuvette. Measurements were carried out at 25 °C under ambient conditions.

The synthesis of FP8[1] and FP18[2] can be found in previous papers.

Supplemental Section 3 Transport Measurement

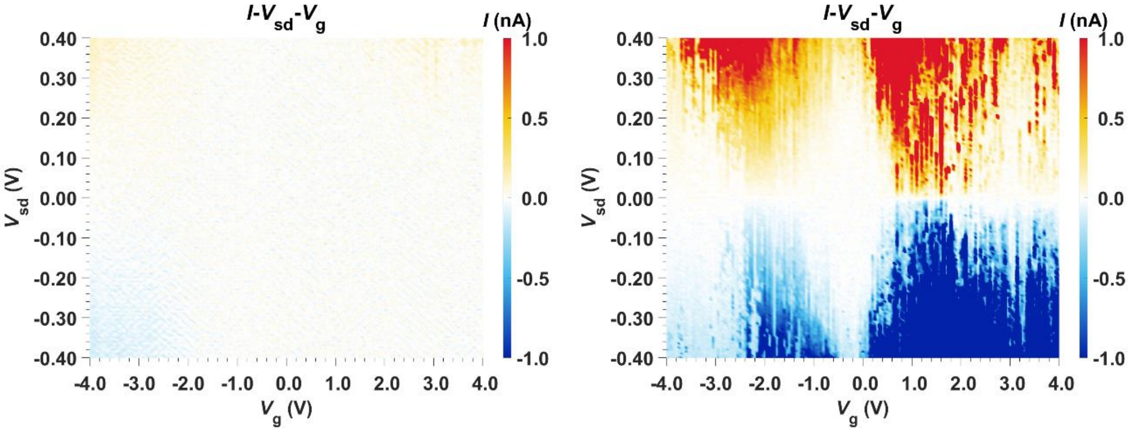

Fig. S3-1 $IV_{sd}V_g$ map before (left) and after (right) **FP8** molecule deposition for device 1 measured at room temperature.

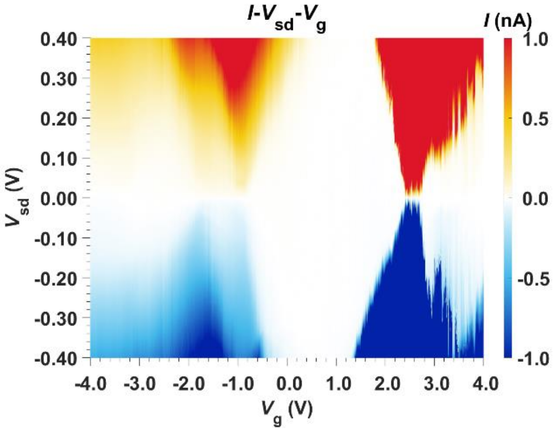

Fig. S3-2 $IV_{sd}V_g$ map for device 1 measured at 4.2 K.

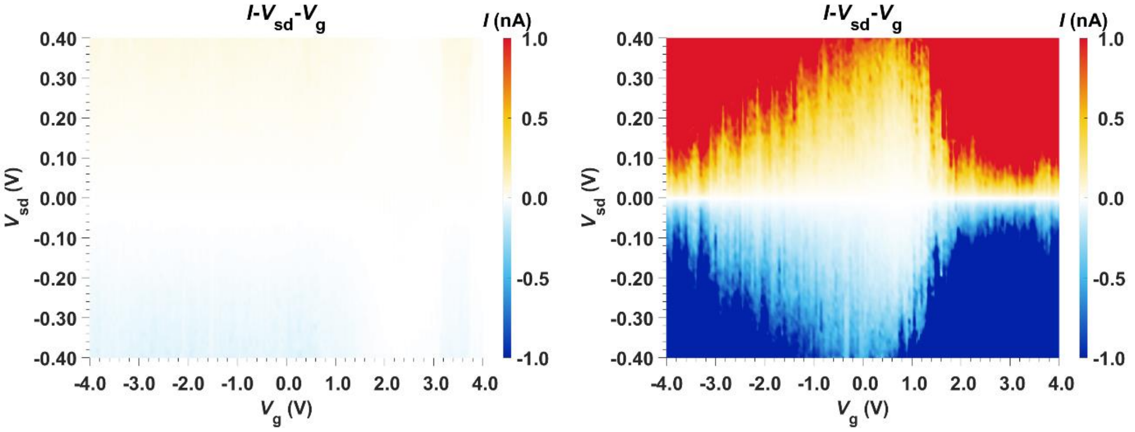

Fig. S3-3 $IV_{sd}V_g$ map before (left) and after (right) **FP18** molecule deposition for device 2 measured at room temperature.

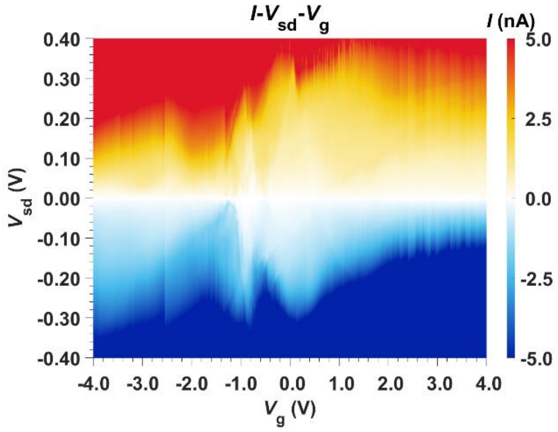

Fig. S3-4 $I V_{\mathrm{sd}} V_{\mathrm{g}}$ map for device 2 measured at 2.8 K.

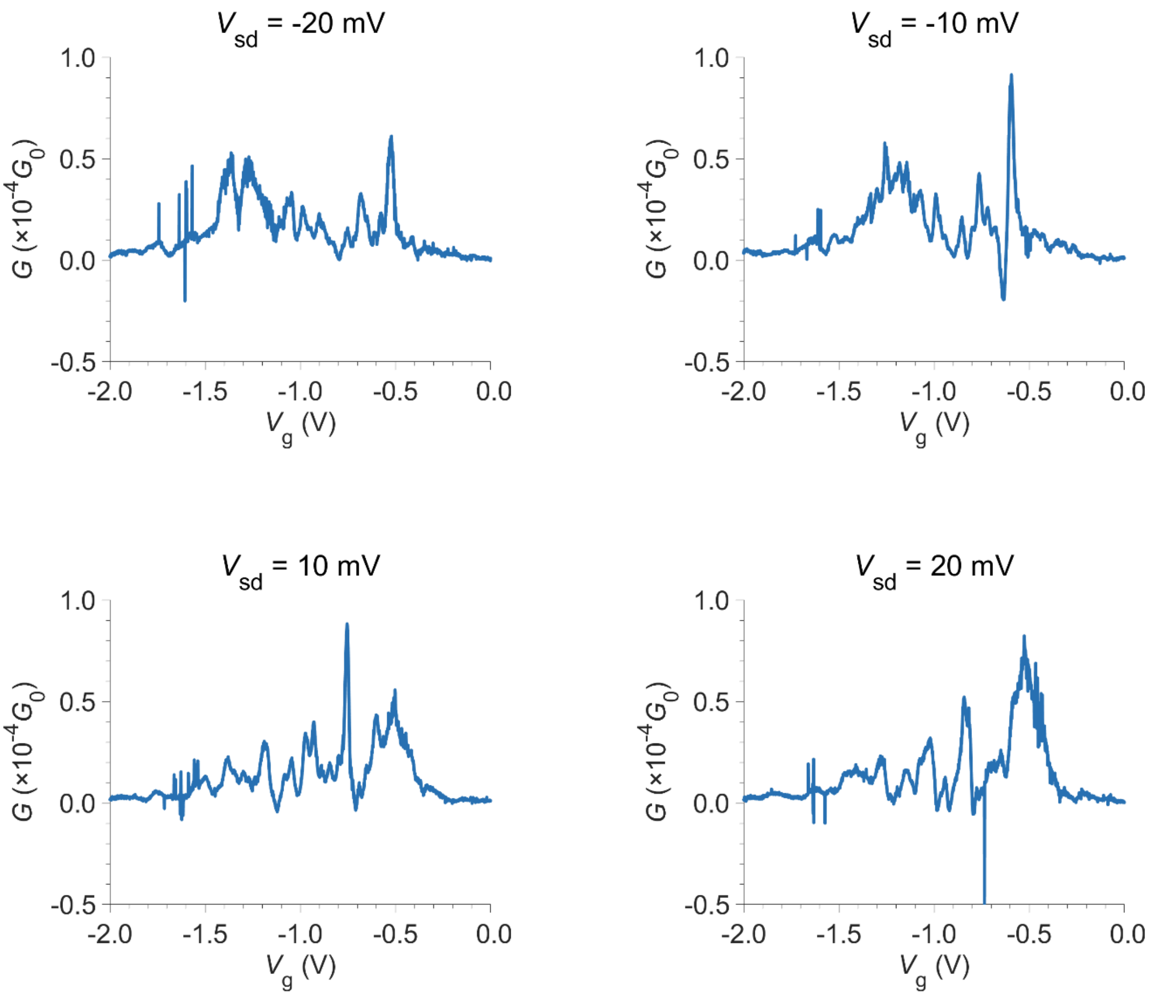

Fig. S3-5 $G V_{\mathrm{g}}$ traces measured at different V_{sd} for device 1.

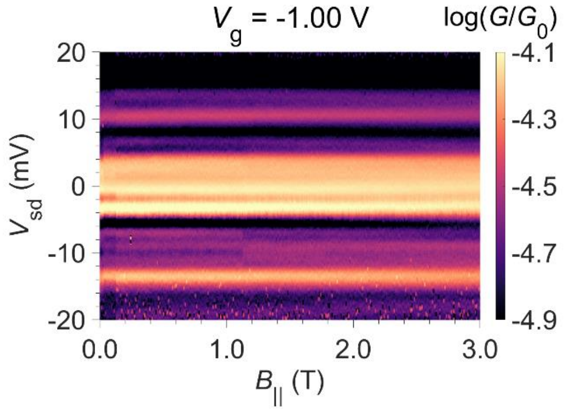

Fig. S3-6 *G* map measured as a function of V_{sd} and magnetic field (*B*) measured at V_g = –1.00 V, showing no conductance fluctuation along changing magnetic field. The applied magnetic field is parallel (||) to transport plane (graphene).

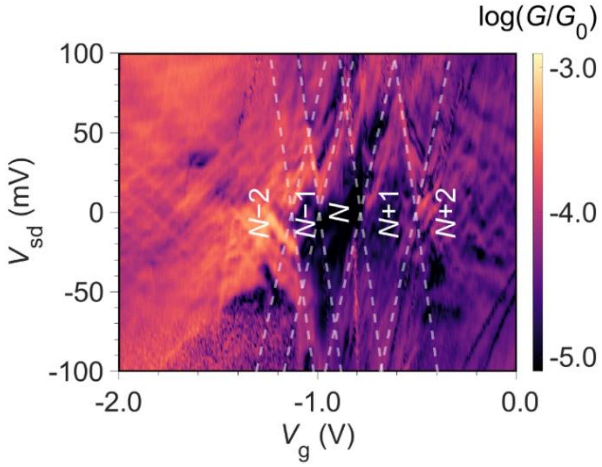

Fig. S3-7 $GV_{sd}V_g$ map (as a ratio to G_0 in log scale) for device 2 measured at 2.8 K. The white dotted lines outline molecular resonances. Charge states are labelled as *N*, *N*+1, *N*+2 etc.

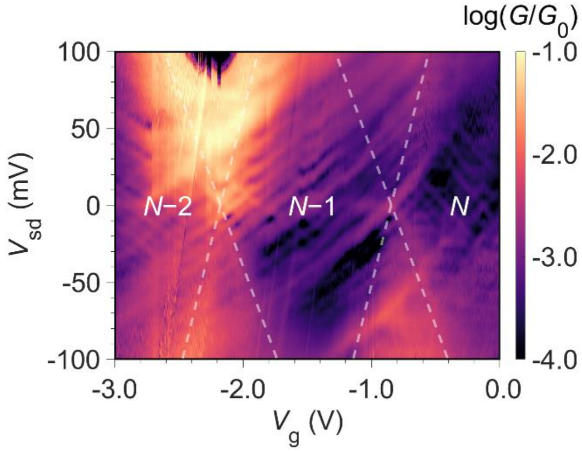

Fig. S3-8 $GV_{sd}V_g$ map for **FP8** device 3 measured at 2.8 K. Data is taken from the same device reported in our previous paper (labelled as Device 2 in the paper).[1] Charge states are labelled as *N*, *N*–1, *N*–2.

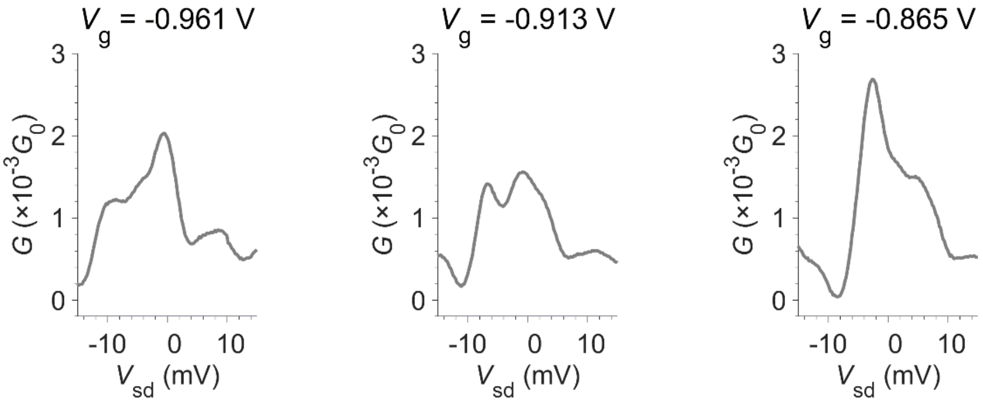

Fig. S3-9 Low bias G-V_{sd} traces around the N–1 / N transition of **FP8** device 3 displaying evolution of Fano line shapes and transmission phase with V_g.

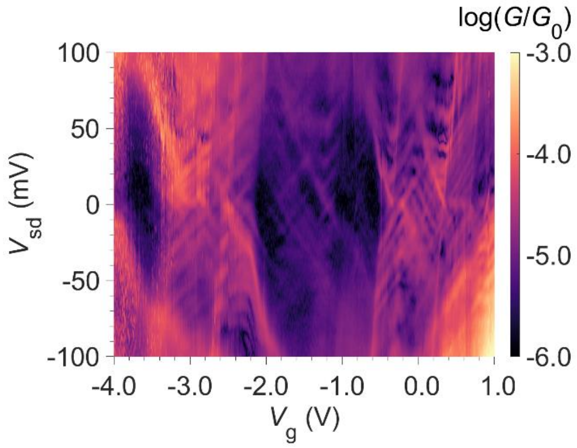

Fig. S3-10 $GV_{sd}V_g$ map for **FP8** device 4 measured at 4.2 K.

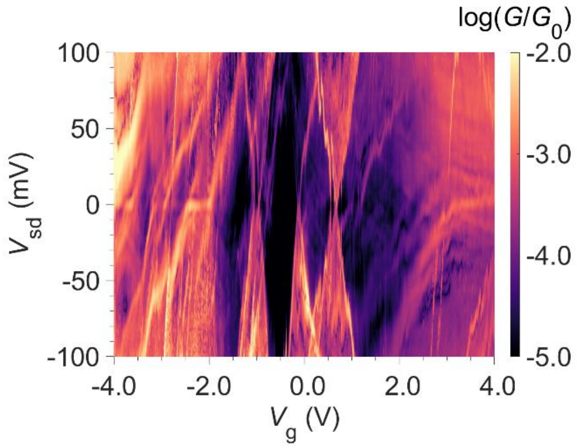

Fig. S3-11 $GV_{sd}V_g$ map for **FP18** device 5 measured at 2.8K.

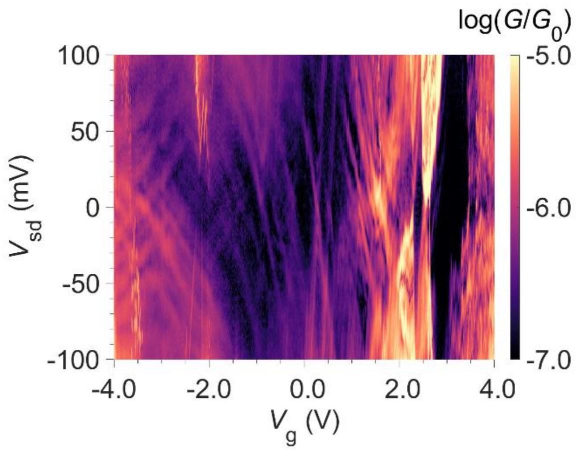

Fig. S3-12 $GV_{sd}V_g$ map for **FP8** device 6 measured at 2.8 K.

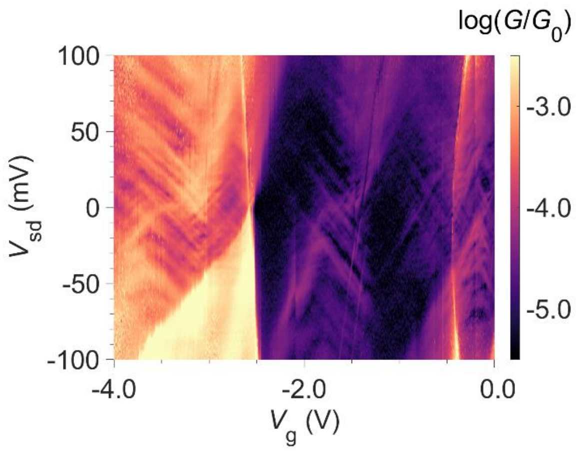

Fig. S3-13 $GV_{sd}V_g$ map for **FP8** device 7 measured at 2.8 K.

Table S3-1. Molecule-electrode coupling constants, Γ, and gate couplings, α_g, for the **FP8** and **FP18** devices studied at 4 K or below. The gate coupling, α_g, is extracted from the slopes of the Coulomb diamonds, and Γ is taken from the FWHM of a Lorentzian fit to a zero-bias Coulomb peak.

Device	***Molecule***	Γ *(eV)*	α_g
***1** (main text)*	FP8	1.40×10^{-2}	0.22
***2** (main text)*	FP18	1.22×10^{-2}	0.31
3	FP8	2.81×10^{-2}	0.11
4	FP8	3.04×10^{-3}	0.09
5	FP18	4.48×10^{-3}	0.20
6	FP18	2.95×10^{-3}	0.17
7	FP18	2.86×10^{-3}	0.24
8	FP8	1.60×10^{-2}	0.11
9	FP18	4.67×10^{-3}	0.22
10	FP18	3.28×10^{-3}	0.21

Supplemental Section 4 Data Analysis

4.1 Discussion about the interference fringes:

The conductance fringes defining the pattern that have a negative gradient are more intense than those with positive gradient, due to asymmetric molecule-graphene coupling, and the slight tilt of the whole pattern is due to an asymmetric potential drop along the device.[3,4]

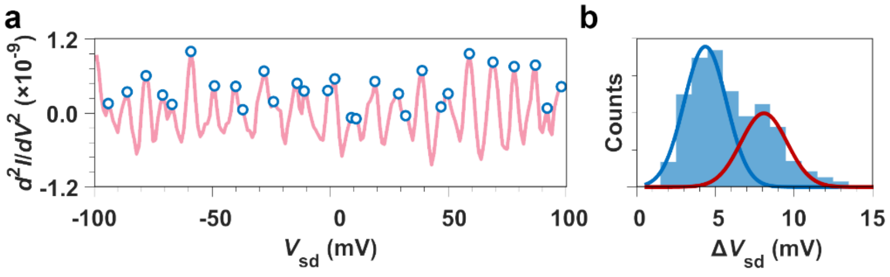

Fig. S4-1 (a) Individual dI^2/dV_{sd}^2-V_{sd} trace at $V_g = -4$ V for device 1 with blue spots marking conductance peaks. (b) The energy spacings between adjacent peaks for each gate voltage are plotted in normalized histograms (total count is 2300) for Figure 1d. Further details can be found in Ref[1]

In a one-dimensional FP cavity, the periodic energy spacings of ~4.4 meV correspond to cavity lengths of $L = hv_F/(2E) = 900$ nm, approximating a Fermi velocity of $v_F = 1.8 \times 10^6$ m/s by adjusting the value for CVD graphene on SiO_2 ($v_F = 2.49 \times 10^6$ m/s) for the larger dielectric constant of HfO_2.[1,5,6]

4.2 Discussion about the temperature-dependent exponential decay of visibility:

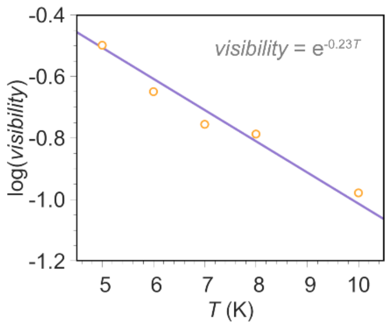

Fig. S4-2 Visibility (in logarithmic scale) as a function of temperature (orange circles) along with a fit (purple line, $R^2 > 0.95$).

The visibility of the FP interference is predicted to decay exponentially: $V(T) = e^{-2\pi^2 k_B T/E}$ where k_B is the Boltzmann constant, T is temperature,[4,7] indicating a blurring of the interference by the thermal broadening of the impinging electrons as the primary mechanism of visibility loss, although the impact of phonons may cause some deviation.[3] $E = 2L/hv_F$ where $2L$ is the length of resonant electron path (double of the cavity length) and v_F is the Fermi velocity of the electrons. An energy of 7.3 meV can be extracted from the fit, which match the scale of the experimental energy spacing 4.1 meV but 70% larger. Or a cavity length of 510 nm can be extracted which also match the experimental cavity length 900 nm but 40% smaller. This might because the oscillation is dominated by more than one resonance condition (e.g. full cavity and half cavity as discussed in [1]) and other resonance conditions have larger energy spacings.

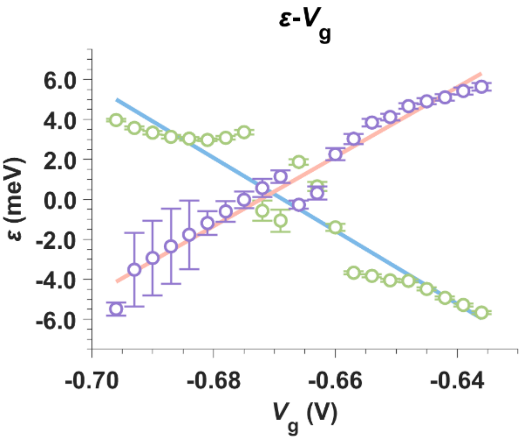

Fig. S4-3 The extraction of $\varepsilon_{\mathrm{FP}}$ (purple) and $\varepsilon_{\mathrm{Mol}}$ (green) from Fano fits, and their linear response (pink line for $\varepsilon_{\mathrm{FP}}$, light blue line for $\varepsilon_{\mathrm{Mol}}$) to gate potentials.

Supplemental Section 5 Simulation

5.1 Equivalent optical cavity interferometer simulation

We introduce an optical model that exhibits the photonic Fano resonance to consolidate our understanding of the interference behaviour observed in our electronic interferometry. In photonics, Fano resonance takes place when two oscillators with different damping rates are weakly coupled, that is, by coupling two resonators with narrow (weakly damped) and broad (strongly damping) spectral lines.[8]

As shown in Figure S5a, our optical structure is formed by two metal-dielectric-metal (MDM) stacked structures. The configuration of each layer is optimized to generate spectrally overlapping resonances with different damping rates. Here the top MDM structure (cavity 1) consists of subwavelength resonators (Au nanodisks, 300 nm in diameter and 25 nm in height) on a substrate with silica (SiO2) as the spacer and 50-nm thick metal Au film underneath, which provides a broadband metasurface absorber[9]. The bottom MDM structure (cavity 2) includes 50 nm thick Au, SiO_2 spacer, and 100 nm thick Au thin-film stack to form the FP cavity. The two cavities share a metal layer (50 nm thick Au) that determines their coupling strength. We perform the finite-difference time-domain (FDTD) simulation (Lumerical Inc.) to investigate optical response of the structure with a plane-wave light incident normally from the top the structure.

Figure S5 (b-c) shows the simulated reflectance from each cavity individually, clearly a presenting a broadband spectral line from cavity 1 and a narrow spectral line from cavity 2. By integrating two cavities together, the Fano resonance is realized in the reflectance, arising from the destructive interference between the spectrally overlapping nanocavities. The reflectance from the whole structure of two coupled resonators is shown in Fig. 5-2, where the asymmetric line shape of the Fano resonance occurs.

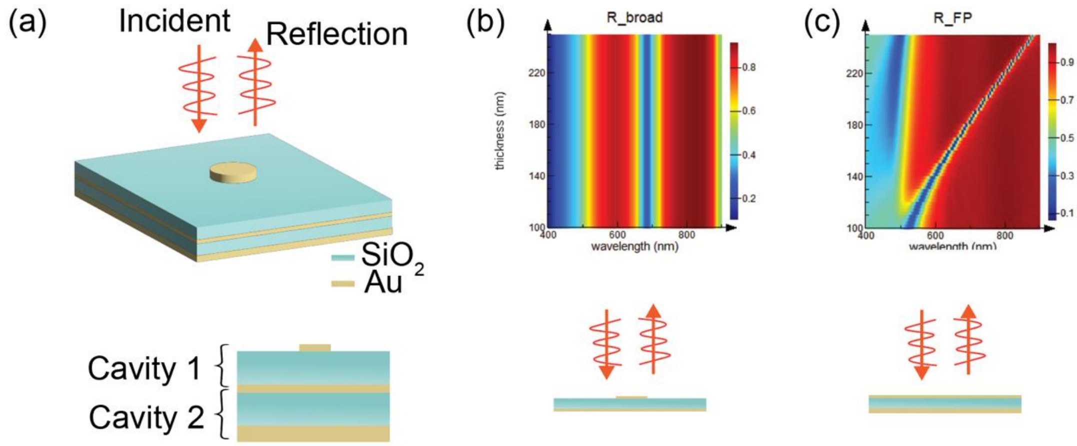

Fig. S5-1 (a) Schematic view of the optical model structure. The simulated reflectance of the broadband nanocavity (b) and the narrowband cavity (c), in terms of spacer thickness and wavelength change.

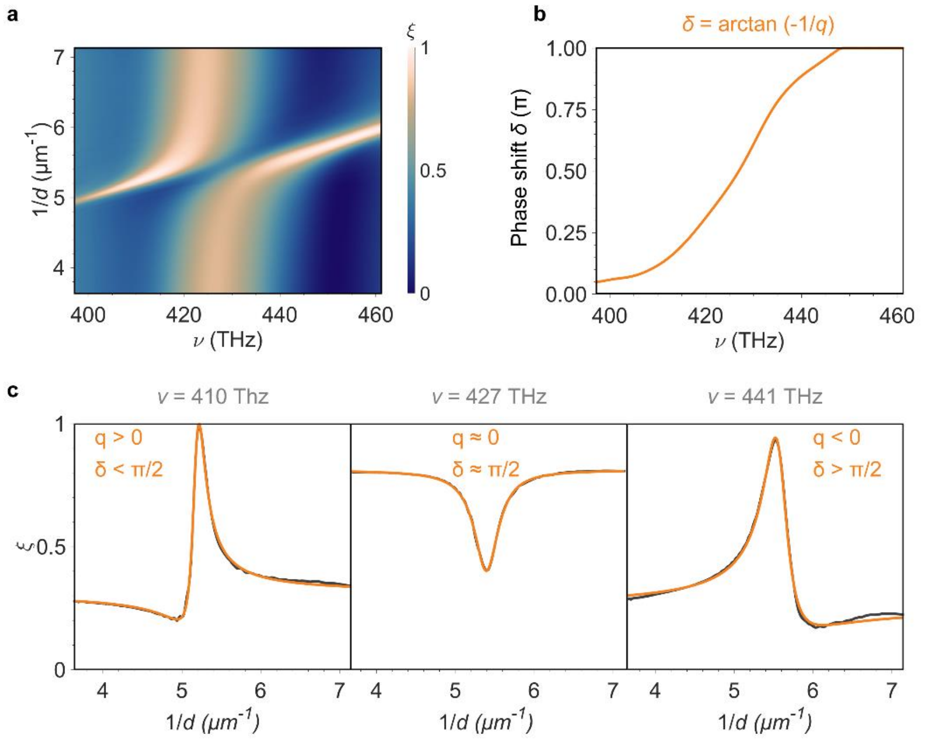

Fig. S5-2 ξ = (1–reflectance) map of optical structure shown in Fig. S5.1, as a function of photon frequency (ν) and inverse cavity length (d^{-1}). The Fano interference is shown by extracted traces through the map and displays the same behaviour as the electronic interferometry. The traces are fitted with Equation 1 (main text), and, as with the electronic measurements, a π phase shift is observed.

5.2 Molecular orbitals

The isolated molecular orbitals were output from a geometry optimization calculation using the B3LYP functional in Gaussian 16.[10] The visualizations of MOs were made in VMD.[11]

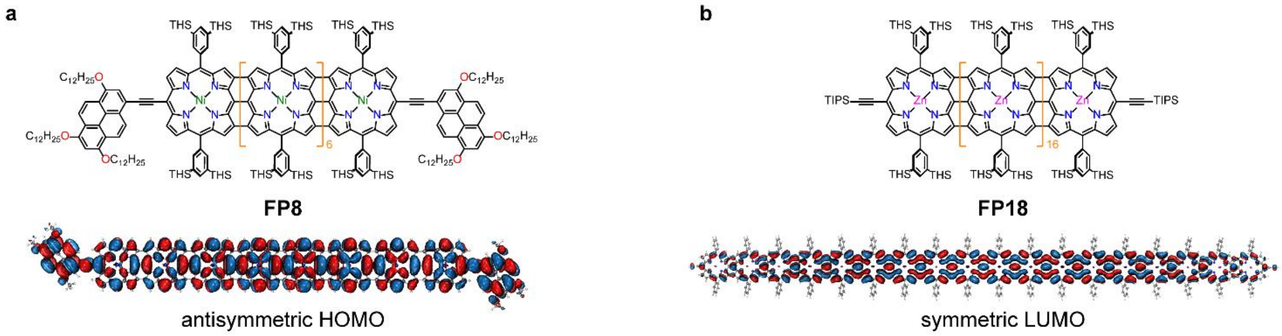

Fig. S5-3 (a) Plot of HOMO of **FP8**, the isovalue is set to 0.005. (b) Plot of LUMO of **FP18**, the isovalue is set to 0.005.

Supplemental Section 6 Schematic Explanation

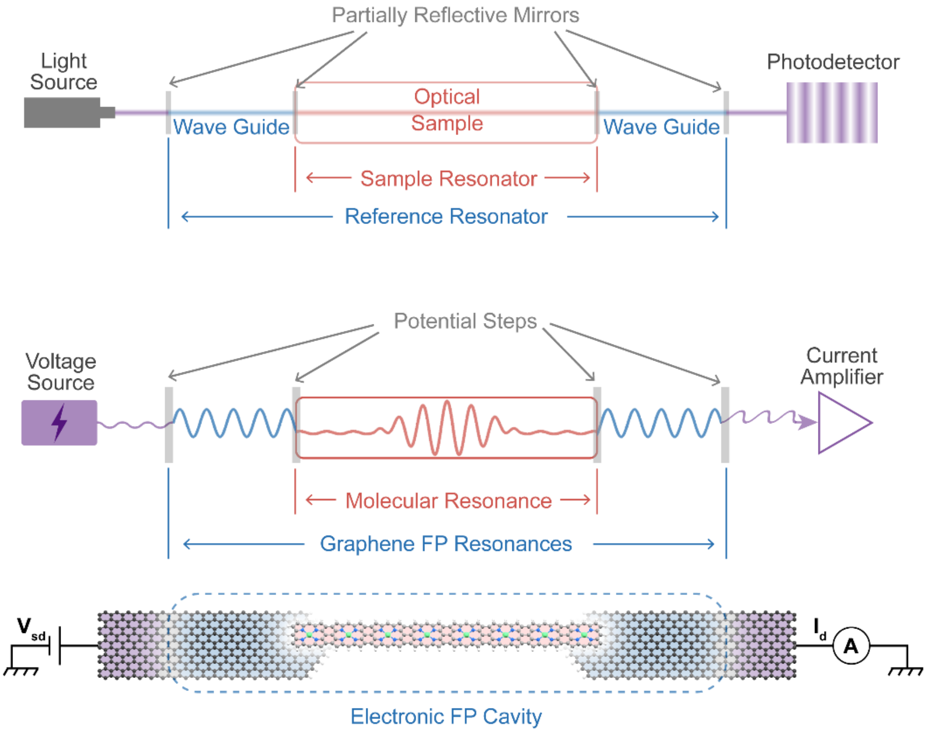

Fig. S6-1 Spatial representation of Figure 1a (not to scale). The graphene FP resonances rely on phase-coherent transport throughout the molecule.[1] The two interfering channels (the molecular resonance and the delocalized state over the FP cavity) partially overlap in real space but are separated in energy space as they have different resonance energies, although this energetic separation can be tuned with the gate voltage, V_g.

Supplemental Reference

1 Chen, Z. *et al.* Phase-Coherent Charge Transport through a Porphyrin Nanoribbon. *J Am Chem Soc* **145**, 15265-15274 (2023). https://doi.org:10.1021/jacs.3c02451

2 Song, S. M. & Cho, B. J. Investigation of interaction between graphene and dielectrics. *Nanotechnology* **21**, 335706 (2010). https://doi.org:10.1088/0957-4484/21/33/335706

3 Deprez, C. *et al.* A tunable Fabry-Perot quantum Hall interferometer in graphene. *Nat Nanotechnol* **16**, 555-562 (2021). https://doi.org:10.1038/s41565-021-00847-x

4 Ronen, Y. *et al.* Aharonov-Bohm effect in graphene-based Fabry-Perot quantum Hall interferometers. *Nat Nanotechnol* **16**, 563-569 (2021). https://doi.org:10.1038/s41565-021-00861-z

5 Ratnikov, P. V. On the dispersion relation of plasmons in a gapless-graphene-based superlattice with alternating Fermi velocity. *JETP Letters* **106**, 810-814 (2017).

6 Hwang, C. *et al.* Fermi velocity engineering in graphene by substrate modification. *Sci. Rep.* **2**, 590 (2012). https://doi.org:10.1038/srep00590

7 McClure, D. T., Chang, W., Marcus, C. M., Pfeiffer, L. N. & West, K. W. Fabry-Perot interferometry with fractional charges. *Phys Rev Lett* **108**, 256804 (2012). https://doi.org:10.1103/PhysRevLett.108.256804

8 Limonov, M. F., Rybin, M. V., Poddubny, A. N. & Kivshar, Y. S. Fano resonances in photonics. *Nat Photonics* **11**, 543-554 (2017). https://doi.org:10.1038/Nphoton.2017.142

9 Jiang, J. Y. *et al.* Colloidal self-assembly based ultrathin metasurface for perfect absorption across the entire visible spectrum. *Nanophotonics* **12**, 1581-1590 (2023). https://doi.org:10.1515/nanoph-2022-0686

10 Gaussian 16 Rev. C.01 (Wallingford, CT, 2016).

11 Humphrey, W., Dalke, A. & Schulten, K. VMD: visual molecular dynamics. *J Mol Graph* **14**, 33-38, 27-38 (1996). https://doi.org:10.1016/0263-7855(96)00018-5
